# Association of Serum High Sensitivity C-Reactive Protein With Pre-diabetes in Rural Population: A Two-Year Cross-Sectional Study

**DOI:** 10.7759/cureus.19088

**Published:** 2021-10-28

**Authors:** Aishwarya Ghule, T. K Kamble, Dhruv Talwar, Sunil Kumar, Sourya Acharya, Anil Wanjari, Shilpa A Gaidhane, Sachin Agrawal

**Affiliations:** 1 Department of Medicine, Jawaharlal Nehru Medical College, Datta Meghe Institute of Medical Sciences (Deemed to be University), Wardha, IND

**Keywords:** body mass index, inflammation, cardiovascular risk factors, high sensitivity c-reactive protein, prediabetes

## Abstract

Introduction: Pre-diabetes is a state of intermediate hyperglycemia. Although it looks benign, pre-diabetes is known to be associated with low-grade inflammation. High sensitivity C-reactive protein (hsCRP) is a sensitive marker to detect low-grade inflammation. Here, we studied whether hsCRP can be used as a biomarker in the early diagnosis of pre-diabetes in a rural population.

Methods: A total of 200 participants, including 100 cases and 100 controls who were age- and gender-matched, were enrolled according to the World Health Organisation criteria for pre-diabetes in this study. All the cases and controls underwent a detailed history, physical examination, anthropometric measurements, and biochemical analysis. The biochemical analysis included blood glucose levels, lipid profile, and hsCRP

Results: The mean hsCRP in pre-diabetics 2.17 ± 0.72 mg/L was significantly higher than controls (0.66 ± 0.22 mg/L; p < 0.0001). High sensitivity CRP was significantly and positively correlated to age, body mass index (BMI), total cholesterol, low-density lipoprotein, cholesterol, and waist-hip ratio.

Conclusion: Raised level of hsCRP was associated with pre-diabetes and also correlated with age, higher BMI, higher cholesterol, higher low-density lipoprotein, and higher waist-hip ratio.

## Introduction

According to International Diabetes Federation, the prevalence of pre-diabetes in the world in 2019 was 8.6% (373.9 million) and was estimated to rise to 9.2% (453.8 million) by 2030 and to 9.5% (548.4 million) by 2045 [1-2]. The National Urban Diabetes Survey reported that the prevalence of pre-diabetes is estimated at 14% in India [3].

The World Health Organisation has defined pre-diabetes as a state of intermediate hyperglycemia. The criteria by Buysschaert and Bergman (2011) for fasting plasma glucose (FPG) is 110 to 125 mg/dL, and for impaired oral glucose tolerance is 140 to 199 mg/dL [1]. Individuals with pre-diabetes are at a higher risk for progression to diabetes and are also known to be associated with an increased risk of cardiovascular disease. Also, pre-diabetes is known to be associated with obesity and a deranged lipid profile [4].

Those with pre-diabetes are usually asymptomatic. So, those with high-risk factors must be screened. Individuals that should be screened include those aged more than 45 years old and Asians with a body mass index of more than 23 kg/m^2^. Others include those less than 45 years but with other risk factors like family history of diabetes, gestational diabetes mellitus, women with high birth weight babies, polycystic ovarian disease, reduced high-density lipoprotein, higher low-density lipoprotein, increased very-low-density lipoprotein, total cholesterol, serum triglycerides, and hypertension. Repeat screening must be done in three years [5-6].

C-reactive protein (CRP) is an acute-phase protein that is increased in infections, inflammatory conditions, and even cancers [7-8]. High sensitivity CRP (hsCRP) is a highly sensitive form of CRP. It is detected by highly sensitive assays and can detect high sensitivity CRP levels sensitively between 0.01 mg/L to 10 mg/L. Thus, these assays can detect even low-grade inflammation in the absence of evident inflammation [7]. Normal high sensitivity CRP levels are less than 10 mg/L. In acute conditions, the levels of high sensitivity CRP rise sharply to more than 10 mg/L. Also, high sensitivity CRP > 1 mg/L is known to be associated with a mild risk of cardiovascular disease, while levels 1-3 mg/L represent moderate risk and > 3 mg/L, high risk [7].

Low-grade inflammation is said to be associated with pre-diabetes. High sensitivity CRP is an indicator of inflammation. Several studies have been reported on the association of high sensitivity CRP and cardiovascular disease [9]. However, very few studies have been done on the relation between high sensitivity CRP and diabetes. Furthermore, even fewer studies have been done on the relation between high sensitivity CRP and pre-diabetes [10].

The other study in the Asian population (involving Indians) done by Sabanyagam et al. in 2011 reported elevated CRP in pre-diabetes and its correlation with several cardiovascular risk factors [11]. Low-grade inflammation was said to be associated with endothelial dysfunction and further causing insulin resistance. Kawamoto et al. in 2011 studied a similar relation in the Japanese population [12]. They concluded that high sensitivity CRP levels increased with the increase in fasting plasma glucose and were not dependent on cardiovascular risk factors [12-14].

As there are few studies on this topic, especially in the Indian population, we intend to study high sensitivity CRP in pre-diabetes in the central Indian rural population.

## Materials and methods

**Figure 1:**
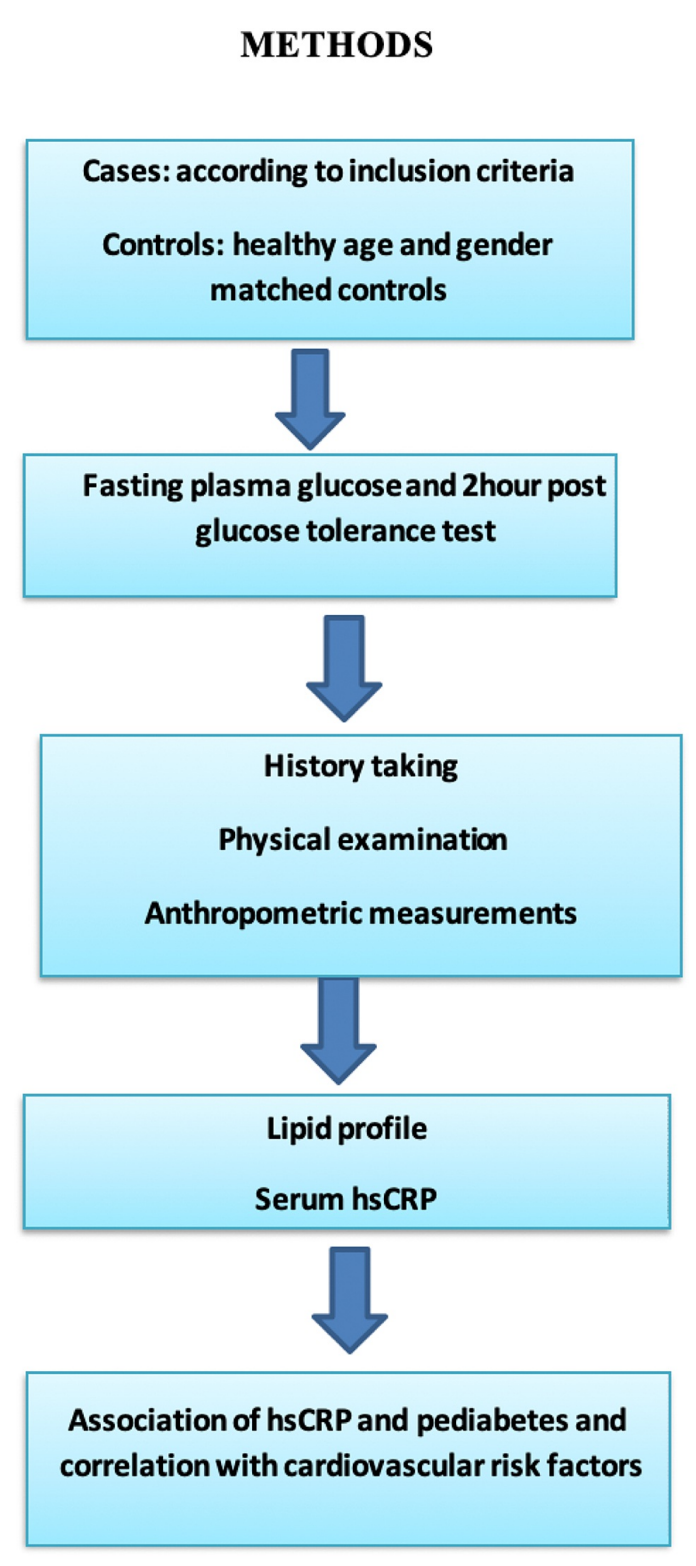
Flowchart showing the method of the study

This cross-sectional study was carried out in Acharya Vinoba Bhave Rural Hospital attached with medical college from October 2018 to August 2020 in central India. Clearance was obtained from the institutional ethics committee with the clearance number as Datta Meghe Institute of Medical Sciences (Deemed to be University)/Institutional Ethics Committee/2018-19/7533 (DMIMS(DU)/IEC/2018-19/7553). All the patients coming to the out-patient department (OPD) who were 18 years and above were taken as cases and controls. Cases were taken according to the World Health Organisation criteria for pre-diabetes. Patients underwent complete history taking and physical examination. All anthropometric measurements like height, weight, body mass index, waist circumference, and waist-hip ratio were taken. Blood investigations included blood glucose levels, lipid profile, and high sensitivity CRP. The hsCRP levels were tested by an ultrasensitive CRP kit; they were measured by the spectrophotometric method by an enzyme-linked immunosorbent assay (ELISA) kit (Turbichem-Hs-CRP kit; Genuine Biosystem, Chennai, India). The test was based on the two-site sandwich enzyme immunoassay principle. The association of high sensitivity CRP in pre-diabetes was studied. Correlation of high sensitivity CRP with various variables was done. A flowchart summarizing the method of study is shown in Figure 1.

Sample size

The National Urban Diabetes Survey estimated the prevalence of pre-diabetes in India at 14% [3]. Our sample size was calculated using this prevalence. The formula used for sample size calculation along with error margin was as follows:

$n = (Zalpha/2)^2 X P (1-P))/d^2$

Where, Zalpha/2 is the level of significance at 5% = 1.96; P = prevalence of pre-diabetes (i.e., 14%, or 0.14); d = desired error of margin (i.e., 7%, or 0.07). So, n = (1.96 × 1.96 × 0.14 × (1-0.14) / 0.07 × 0.07 = 94.39. Thus, we chose a sample size of 100 for the study, i.e., 100 pre-diabetic patients as cases and 100 controls.

Inclusion Criteria

All patients coming to the OPD in the age group of 18 and above, diagnosed and fulfilling the WHO criteria for pre-diabetes, were included as cases. The WHO criteria of pre-diabetes, as stated by Buysschaert & Bergman in 2011, are (a) a fasting serum glucose level between 110 mg/dL and 125 mg/dL and/or (b) plasma glucose levels two-hours after 75 gm oral glucose tolerance test (OGTT) between 140 mg/dL and 199 mg/dL [1]. The controls were age- and sex-matched.

Exclusion Criteria

We excluded the patients who had the following: (a) acute inflammatory conditions, infections, and tissue damage like burns or injuries, pneumonia, febrile conditions, acute pancreatitis, myocardial infarction; (b) chronic infections like tuberculosis; (c) malignancies; (d) chronic inflammatory diseases such as systemic lupus erythematosus, vasculitis, inflammatory bowel disease, and rheumatoid arthritis; and (e) hormone replacement therapy. We also excluded post-operative patients and patients taking medications like statins and fibrates, where high sensitivity CRP levels are found to be decreased.

Statistical analyses

Descriptive and inferential statistics were used for analyses. Chi-square test was used for testing the distribution of age, gender, body mass index, waist-hip ratio, fasting blood sugar, oral glucose tolerance test, total cholesterol, high-density lipoprotein, low-density lipoprotein, very low-density lipoprotein, and high sensitivity CRP in the cases and controls. Pearson’s correlation coefficient test was used to test the statistical significance of the correlation between high sensitivity C-reactive protein and age, BMI, waist circumference, waist-hip ratio, total cholesterol, high-density lipoprotein, or low-density lipoprotein in pre-diabetics. The software used in the analyses were SPSS Statistics version 24.0 (IBM Corp., Armonk, NY) and GraphPad Prism version 7.0 (GraphPad Software, Inc., San Diego, CA). P-value of < 0.05 was considered significant.

## Results

Out of 100 cases, the majority were in the age group 40 to 60 years, i.e., 48 (48%), and the average age was 51.92 ± 9.63 years. Out of 100 controls, the majority were in the age group 40 to 60 years,i.e., 49 (49%), and the mean age was 49.35 ± 13.52 years. Chi-square was 1.54, and p-value was 0.12, thus not significant. Therefore, the cases and controls were age-matched. All other baseline characteristics are shown in Table 1.

**Table 1:** Baseline characteristics of the study population Abbreviations: NS, not significant; S, significant

Variables	Group						Chi-Squared Test	
	Case		Control		Total		χ2	p-value
	N=100	%	N=100	%	N	%		
Age, Mean ± SD	51.92 ± 9.63		49.35 ± 13.52		50.63 ± 11.57		t=1.54	0.12, NS
Gender								
Male	65	65	67	67	132	66	0.08	0.76, NS
Female	35	35	33	33	68	34		
Body Mass Index								
<18.5 kg/m^2^	3	3	3	3	6	3		
18.5-22.9 kg/m^2^	72	72	79	79	151	75.5		
23.0-27.5 kg/m^2^	19	19	14	14	33	16.5		
>27.5 kg/m^2^	6	6	4	4	10	5		
Body Mass Index, Mean ± SD	24.50 ± 3.83		23.66 ± 2.70		24.08 ± 3.33		1.48	0.68, NS
Waist circumference								
High	23	23	16	16	39	19.5		
Normal	77	77	84	84	161	80.5		
Waist Circumference, Mean ± SD	81.6 ± 10.21		80.41 ± 6.17		81.00 ± 8.44		1.56	0.21, NS
Waist-Hip Ratio								
High	21	21	8	8	29	14.5		
Normal	79	79	92	92	171	85.5		
Waist-Hip Ratio, Mean ± SD	0.84 ± 0.09		0.81 ± 0.06		0.83 ± 0.08		2.73	0.007, S

The mean value of high sensitivity CRP for cases was 2.17 ± 0.72 mg/L. The mean value of high sensitivity CRP for controls was 0.66 ± 0.22 mg/L. High sensitivity CRP for cases was higher than controls. The chi-square value was 152.693. The p-value was < 0.001, i.e., significant, as shown in Table 2.

**Table 2:** Distribution of cases and controls according to high sensitivity C-reactive protein (hsCRP)

hsCRP	Group		p value
	Case (n = 100)	Control (n =100)	
<1 mg/L, N (%) (low risk)	6 (6%)	87 (87%)	<0.001
1-3 mg/L, N (%) (intermediate risk)	87 (87%)	13 (13%)	<0.001
≥3 mg/L, N (%) (high risk)	7 (7%)	0 (0%)	<0.001
hsCRP (mg/L), Mean ± SD	2.17 ± 0.72	0.66 ± 0.22	

**Figure 2:**
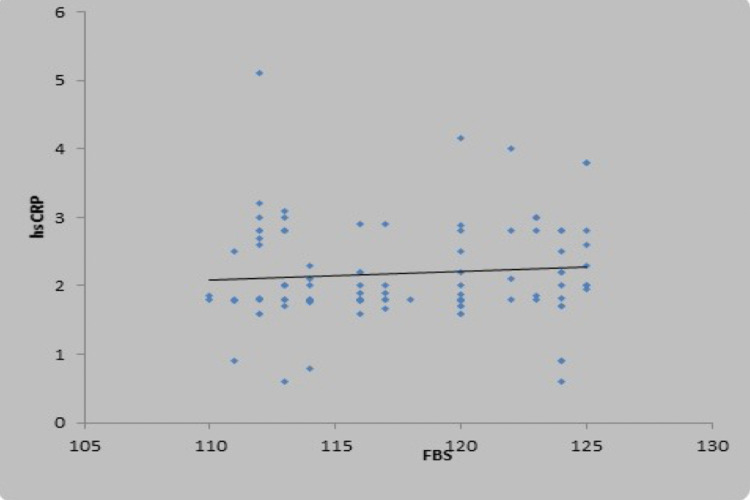
Association between high sensitivity C-reactive protein (mg/L) and fasting blood sugar in cases Abbreviations: hsCRP, high sensitivity C-reactive protein; FBS, fasting blood sugar

**Figure 3:**
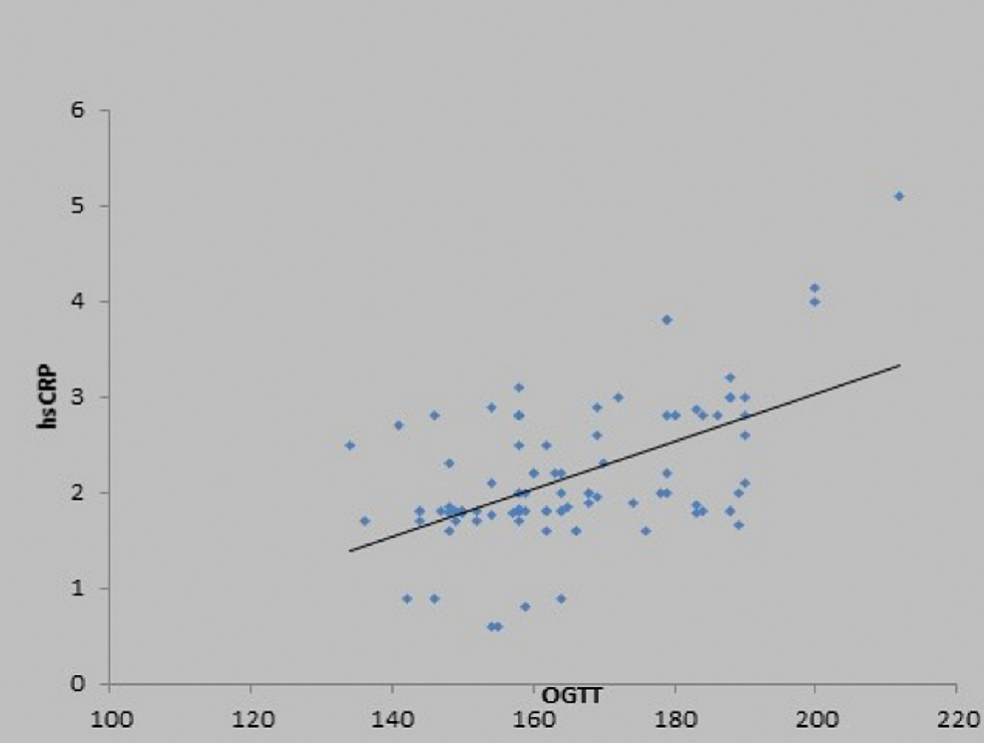
Association of high sensitivity C-reactive protein (mg/L) and oral glucose tolerance test in cases Abbreviations: hsCRP, high sensitivity C-reactive protein; OGTT, oral glucose tolerance test

**Figure 4:**
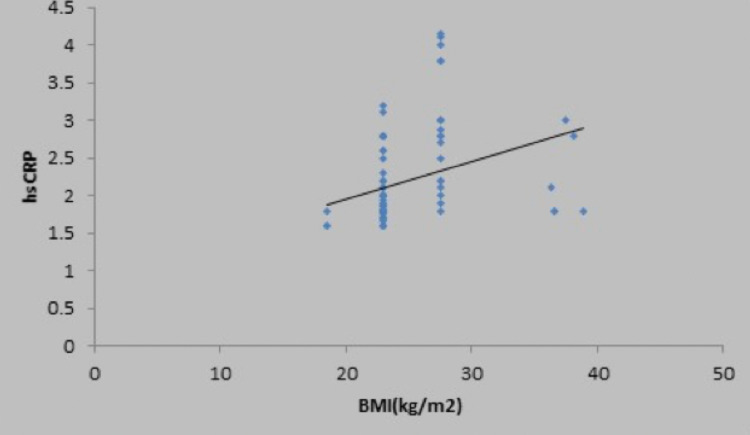
Association between high sensitivity C-reactive protein (mg/L) and body mass index in cases Abbreviations: hsCRP, high sensitivity C-reactive protein; BMI, body mass index

**Figure 5:**
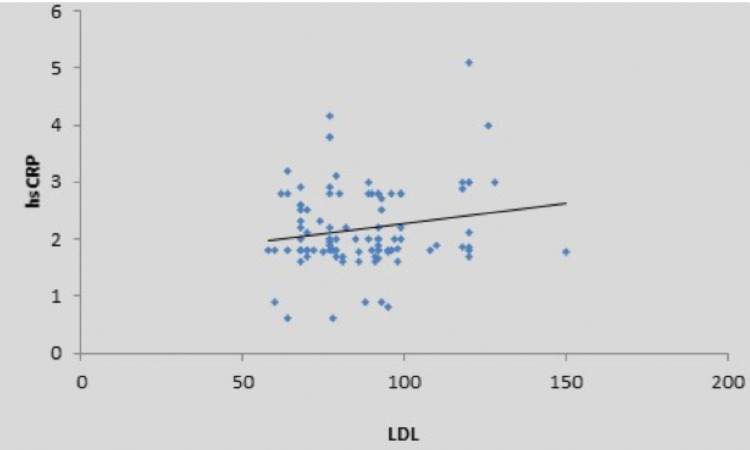
Association between high sensitivity C-reactive protein (mg/L) and low-density lipoprotein in cases Abbreviations: hsCRP, high sensitivity C-reactive protein; LDL, low-density lipoprotein

**Figure 6:**
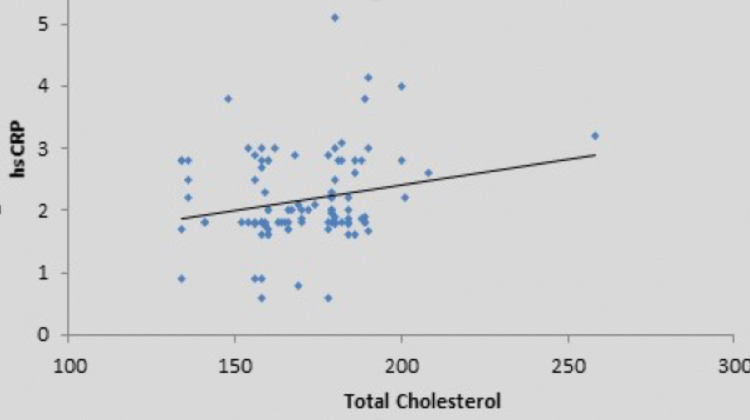
Association between high sensitivity C-reactive protein (mg/L) and total cholesterol in cases Abbreviations: hsCRP, high sensitivity C-reactive protein

There was a weak positive correlation between fasting blood sugar (mg/dL) and high sensitivity CRP (mg/L), and this correlation was not statistically significant (rho = 0.15, p = 0.132) (Figure 2). There was a moderate positive correlation between oral glucose tolerance test (mg/dL) and high sensitivity CRP (mg/L), and this correlation was statistically significant (rho = 0.47, p = <0.001), as shown in the scatter diagram in Figure 3. There was also a positive correlation of high sensitivity CRP with body mass index, low-density lipoprotein, and total cholesterol, as shown in Figures 4-6 in all cases of pre-diabetes.

## Discussion

In this study, we found high sensitivity CRP as a definite predictor of inflammation in pre-diabetes, which was the primary outcome. It was also found that high sensitivity CRP was directly proportional to the cholesterol and low-density lipoprotein levels which were the risk factor of pre-diabetes, taken as a secondary outcome.

In our study, out of 100 cases, the mean ± SD high sensitivity CRP was 2.17 ± 0.72 mg/L and in the control group, it was 0.66 ± 0.22 mg/L. Similarly, Lin et al. (2009) reported a positive association between elevated CRP and pre-diabetes among the Chinese Han population [13]. The mean ± SD of high sensitivity C-reactive protein in cases was 2.44 ± 3.341 mg/L and in controls was 18 ± 2.48 mg/L. It was significantly higher in cases than controls [13]. Moreover, Sabanyagamet et al. (2011) reported a study of two Asian cohorts, SP2 and SiMES [11]. In the SP2 cohort, the mean ± SD of CRP in cases was 4.2 ± 9.0 mg/L and in controls was 2.3 ± 5.1 mg/L, and was statistically significant, i.e., pre-diabetics had significantly higher levels of high sensitivity C-reactive protein than controls. However, in the SiMES cohort, the mean ± SD of CRP levels in cases was 3.7 ± 7.6 mg/L and in controls was 4.2 ± 8.5 mg/L. This difference was statistically non-significant, i.e., the pre-diabetics did not have significantly higher CRP levels than controls [11]. Similarly, Jaiswal et al. (2012) stated that those with IGT (cases) and IFG (cases) had higher median levels of hsCRP, i.e., 2.32 mg/L and 2.20 mg/L, respectively, than those with normal blood glucose levels who had the median high-sensitivity C-reactive protein value of 1.64 mg/L [10]. Moreover, our results were in accordance with a study done by Ravish H et al. (2015). The mean ± SD hsCRP in pre-diabetics was 4.77 ± 3.95 mg/L and in controls was 2.35 ± 2.64 mg/L. They found that mean values of high sensitivity C-reactive protein showed a significant increase among pre-diabetic patients when compared with normal controls.(14)

Inflammatory marker hsCRP was found to be related to the metabolic profiles and was found to be a good prognostic marker of cardiovascular complications in type 2 diabetic patients without clinical atherosclerotic manifestations by various studies [15,16]. This indicates the significance of hsCRP in predicting cardiovascular complications in otherwise healthy-looking patients with raised blood glucose levels.

There was a strong positive correlation between BMI and high sensitivity CRP, and this correlation was statistically significant (rho = 0.67, p = <0.001) in our study. However, Sabanayagam et al.. (2011) reported that the association between CRP and pre-diabetes was stronger among those with BMI < 25 kg/m^2,^ and it was statistically significant [6,11]. Similarly, Jaiswal et al. (2012) reported in their study a significant increase in high sensitivity CRP levels in those with impaired glucose tolerance, and it was statistically significant in those with body mass index < 23 kg/m^2^ [10]. In our study, there was a significant correlation of high sensitivity CRP with the various parameters such as serum total cholesterol, LDL, and waist-hip ratio. However, in a study by Jaiswal et al. (2012), there was a significant correlation between high-density lipoprotein < 50 mg/dL and high sensitivity C-reactive protein levels in pre-diabetes. Those with less (< 50 mg/dL) high-density lipoprotein levels were likely to have higher high sensitivity CRP [6,10].

Limitations

This study was conducted using a small-sized sample, and therefore it cannot be generalized; hence further studies are required. Also, glycosylated hemoglobin (HbA1C) could not be measured in our study due to the cost constraints of a rural setup. This study was conducted in a rural area of central India in Maharashtra, and hence it has regional limitations.

## Conclusions

High sensitivity C-reactive protein, which is a marker of inflammation, was also found to be correlated with deranged lipid profile and oral glucose tolerance test. We conclude that high sensitivity C-reactive protein can be used as an early predictor of inflammation in pre-diabetes and can be a marker of underlying deranged sugar levels and lipid profile in pre-diabetics unaware of their health status.
